# Optimal coordinated control of hybrid AC/VSC-HVDC system integrated with DFIG via cooperative beetle antennae search algorithm

**DOI:** 10.1371/journal.pone.0242316

**Published:** 2020-11-18

**Authors:** Junfang Hao, Jinhai Huang, Ailing Zhang, Hongjie Ai, Qun Zhang, Bo Yang

**Affiliations:** 1 XJ Electric Co., Ltd., Xuchang, China; 2 Faculty of Electric Power Engineering, Kunming University of Science and Technology, Kunming, China; Huazhong University of Science and Technology, CHINA

## Abstract

Nowadays, with the significant integration of various renewable energy, hybrid alternating current/ voltage source converter based high voltage direct current (AC/VSC-HVDC) system integrated with doubly-fed induction generator (DFIG) has achieved rapidly development in smart grid. A proper control system design for hybrid AC/VSC-HVDC system plays a very crucial role for a reliable and effective power transmission. Hence, this paper designs a novel cooperative beetle antenna search (CBAS) algorithm for optimal coordinated control of hybrid AC/VSC-HVDC system integrated with DFIG. Compared with original beetle antennae search (BAS) algorithm, CBAS algorithm can significantly improve searching efficiency via an efficient cooperation with a group of multiple beetles instead of a single beetle. Particularly, CBAS algorithm can effectively escape from local optimums thanks to its dynamic balance mechanism, which can maintain appropriate trade-off between global exploration and local exploitation. Moreover, three case studies are undertaken to validate the effectiveness and superiorities and effectiveness of CBAS algorithm compared against that of other traditional meta-heuristic algorithms. Especially, the average results of fitness function acquired by CBAS algorithm is merely 46.05%, 41.18%, and 47.82% of that of PSO, GA, and BAS algorithm, respectively.

## 1. Introduction

With the rapid development and wide application of renewable energy [1], new materials [2], and advanced power electronics [3], requirements for higher power supply quality, reliability, and operation efficiency are ever-increasing in the past decade. High voltage direct current (HVDC) transmission technology owns elegant merits of flexible operation [4], fast power regulation [5], high reliability [6], and improved system transient stability [7]. It has achieved wide application in long-distance power transmission [8], asynchronous interconnection [9], and submarine power transmission [10]. Particularly, voltage source converter based high voltage direct current (VSC-HVDC) system shows higher superiorities in many aspects compared with that of current source converter based HVDC (CSC-HVDC) system [11,12], such as higher flexibility. VSC-HVDC transmission technology is an ideal transmission method for large-scale grid-connected wind farms. Such grid-connected methods can better improve the transmission capacity of wind power and system operation stability [13]. In recent years, hybrid alternating current/ voltage source converter based high voltage direct current (AC/VSC-HVDC) system is envisaged as an important part of renewable energy transmission, e.g., wind energy integration such as doubly fed induction generator (DFIG), which can enhance the system stability and reliability. Needless to say, design of a proper control system is critical to an effective operation of hybrid AC/VSC-HVDC system.

In general, proportional-integral (PI) or proportional-integral-differential (PID) controllers are conventionally adopted by HVDC systems thanks to its high reliability and easy implementation. However, their gains are determined through local linearization and generally tuned through trial-and-error. When power system is subject to large disturbances and deviates far from the operation point, an effective control can hardly be achieved by PI/PID controllers [14,15]. With the development of nonlinear control theory, nonlinear control was first successfully applied in converter control of affine nonlinear model of HVDC system in literatures [16,17]. However, feedback linearization method requires accurate system parameters and measurable states, which lacks of robustness against various changes in parameters and models [18]. Thus, an inverse system method was proposed to establish a third-order non-affine and nonlinear DC model based on feedback linearization, upon which a combined commutation controller has been designed to satisfy the pole configuration requirements while the stability of closed-loop system is proved [19]. Besides, an inverse system method of multivariable nonlinear control was adopted to design a feedback linearization and quadratic optimal combination controller, which can improve the stability of converter station and prevent commutation failure [20]. However, the aforementioned controllers still lack of high flexibility and reliability under different operation conditions and disturbances.

On the other hand, various artificial intelligence methods, such as fuzzy control, robust control, neural network (NN), and meta-heuristic algorithms have been widely used in HVDC transmission control systems. A sequential decentralized control technology was presented to design a fuzzy logic controller [21], which owns benefits of simple structure and can effectively improve damping characteristics of HVDC system. Besides, literature [22] designed a high-gain and low-frequency controller for inverter side connected to weak AC system based on *H*_∞_ control theory, which shows high robustness to uncertain AC system parameters and operation conditions. Besides, PI controller and NN controller based linearized HVDC systems are reported [23], which demonstrates that NN controller is superior to PI controller in terms of damping and robustness. Furthermore, a new interactive teaching-learning optimizer (ITLO) for VSC-HVDC systems with integration of offshore wind farm was designed, which can dramatically enhance controlling performance at different operating scenarios via optimal tuning of parameters of eight interactive PI loops [24].

Generally speaking, in a hybrid AC/VSC-HVDC system, VSC-HVDC system can effectively adjust power factor and enhance system stability, while the control of AC system such as excitor control, power system stabilizer (PSS), and reactive voltage control can also affect VSC-HVDC system. Hence, coordinated control between them becomes a very important task, e.g., a dual-mode control strategy for AC/VSC-HVDC hybrid transmission system control with wind farm integration was proposed [25], in which an improved direct-current vector control approach is employed for grid-side VSC control. Moreover, literature [26] proposed a novel coordinated control technique to achieve emulation of synchronous generators inertia. Meanwhile, combination of direct feedback linearization and linear optimal theory was applied to design a nonlinear optimal control rule for coordination of HVDC and generator excitor [27]. Furthermore, based on small interference linearization model, decentralized control and genetic algorithm (GA) are combined to achieve an optimal coordination of multiple controller parameters in hybrid HVDC systems [28].

However, structure of the above control schemes for hybrid AC/VSC-HVDC system are usually complex, which hinders its applications in practice. In order to realize an optimal control design of hybrid AC/VSC-HVDC system integrated with DFIG, this paper designs a novel bio-inspired meta-heuristic algorithm called cooperative beetle antennae search (CBAS) algorithm, to achieve optimal gains tuning of PSS gains of synchronous generator, PI gains of VSC-HVDC system, and PI gains of DFIG under various operation scenarios. Compared to the original beetle antennae search (BAS) [29,30] algorithm which mimicking searching mechanism of long-horn beetles, a cooperative group of multiple beetles instead of a single beetle is introduced by CBAS algorithm to realize a dynamic balance between local exploitation and global exploration, upon which an optimal control gains tuning are simultaneously achieved for hybrid AC/VSC-HVDC system integrated with DFIG.

The rest of this paper is organized as: Section 2 presents the modelling of hybrid AC/VSC-HVDC system integrated with DFIG; Then, basic principle of CBAS algorithm is introduced in Section 3; Section 4 elaborates detailed design of CBAS algorithm based optimal coordinated control for hybrid AC/VSC-HVDC system integrated with DFIG; Section 5 undertakes three case studies to validate its effectiveness. At last, conclusions are presented in Section 6.

## 2. Hybrid AC/VSC-HVDC system integrated with DFIG modelling

**Fig 1:**
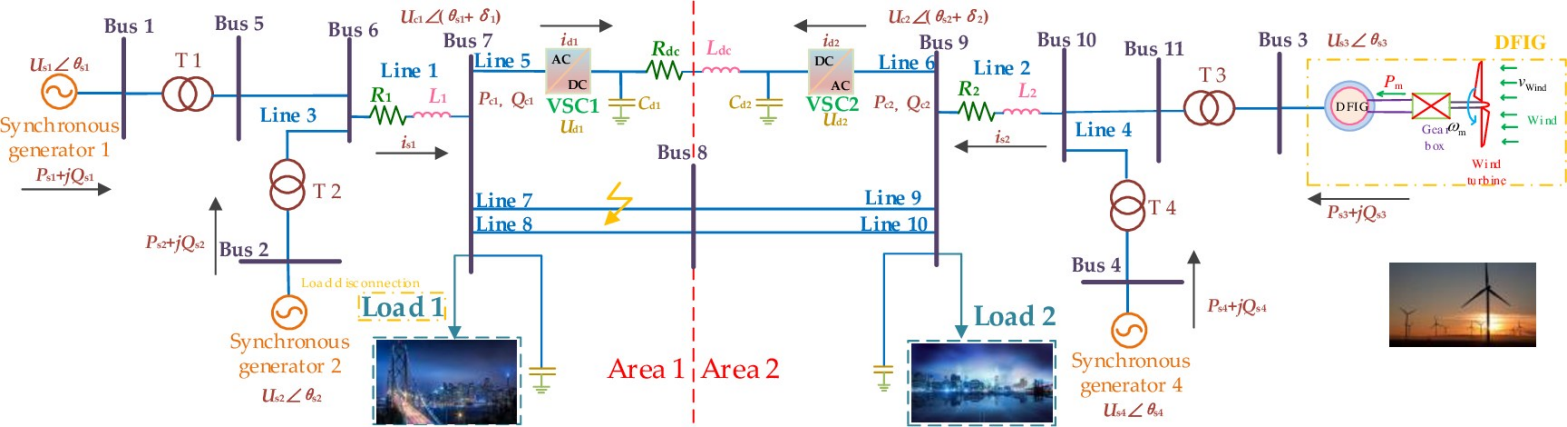
Hybrid AC/VSC-HVDC system integrated with DFIG structure.

Hybrid AC/VSC-HVDC system integrated with DFIG is illustrated in Fig 1 based on typical 4 machines 11 bus (4M11B) systems, which includes three synchronous generators (#1, #2, #4) and one DFIG (#3). Meanwhile, VSC-HVDC is connected between bus 7 (rectifier side) and bus 9 (inverter side) and operates in parallel with two AC lines to transmit power. Here, *R*_1_ and *R*_2_ represent equivalent resistances of coupling transformer and phase reactor, respectively; *L*_1_ and *L*_2_ represent equivalent inductances of coupling transformer and phase reactor, respectively; *U*_s*i*_∠*θ*_s*i*_(*i* = 1,⋯,4) and *U*_c*i*_∠(*θ*_s*i*_+*δ*_*i*_)(*i* = 1,2) represent generator voltages and voltages of point of common coupling (PCC); *P*_s*i*_ and *Q*_s*i*_ denote active and reactive power of AC system; *P*_c*i*_ and *Q*_c*i*_ are active and reactive power of VSC-HVDC system; *i*_s*i*_ means the current flowing from AC system to VSC; *R*_dc_ and *L*_dc_ denote resistance and inductance of DC line, respectively.

### 2.1 Synchronous generator model

The *n*th machine in a multimachine power system with *n* machines represents the reference machine, which is expressed by [31]

$$\left\{ \begin{array}{l} {\dot{\delta }}_{i}=\omega_{i}-\omega_{0}\\ {\dot{\omega }}_{i}=\frac{\omega_{0}}{2H_{i}}(P_{mi}-\frac{D_{i}}{\omega_{0}}(\omega_{i}-\omega_{0})-P_{ei})\\ {\dot{E}}_{qi}^{\prime }=\frac{1}{T_{d0i}}(u_{fdi}-E_{qi}),\;i=1,2,\cdots ,n \end{array}\right.$$ (1)

with

$$\left\{ \begin{array}{l} E_{qi}=E_{qi}^{\prime }+(x_{di}-x_{di}^{\prime })I_{di}\\ P_{ei}=E_{qi}^{\prime }I_{qi}+{E_{qi}^{\prime }}^{2}G_{ii}\\ I_{di}=-\sum_{j=1,j\neq i}^{n}E_{qj}^{\prime }Y_{ij}\cos\left(\delta_{i}-\delta_{j}\right)\\ I_{qi}=-\sum_{j=1,j\neq i}^{n}E_{qj}^{\prime }Y_{ij}\sin\left(\delta_{i}-\delta_{j}\right) \end{array}\right.$$ (2)

where the meaning of all variables/parameters contained in Eq (1) and Eq (2) can be referred to literature [31].

### 2.2 DFIG model

The mechanical power that the wind turbine can capture is given as follows [32]

$$P_{m}=\frac{1}{2}\rho \pi R^{2}C_{p}(\lambda ,\beta )v_{wind}^{3}$$ (3)

where *ρ* means the air density; *R* denotes wind turbine’s radius; and *v*_wind_ stands for the wind speed; *C*_p_(*λ*,*β*) represents the power coefficient, in which *λ* can be expressed as

$$\lambda =\frac{\omega_{m}R}{v_{wind}}$$ (4)

where *ω*_m_ represents wind turbine’s rotational speed. Considering the characteristics of wind turbines, a generic equation used to describe *C*_p_(*λ*,*β*), as follows

$$C_{p}(\lambda ,\beta )=c_{1}\left(\frac{c_{2}}{\lambda_{1}}-c_{3}\beta -c_{4}\right)e^{-\frac{c_{5}}{\lambda_{1}}}+c_{6}\lambda$$ (5)

with

$$\frac{1}{\lambda_{i}}=\frac{1}{\lambda +0.08\beta }-\frac{0.035}{\beta^{3}+1}$$ (6)

where coefficients *c*_1_ to *c*_6_ are selected as: *c*_1_ = 0.5176, *c*_2_ = 116, *c*_3_ = 0.4, *c*_4_ = 5, *c*_5_ = 21, and *c*_5_ = 0.0068.

The DFIG dynamics can be expressed by

$$\left\{ \begin{array}{l} \frac{di_{qs}}{dt}=\frac{\omega_{b}}{L_{s}^{\prime }}(-R_{1}i_{qs}+\omega_{s}L_{s}^{\prime }i_{qs}+\frac{\omega_{r}}{\omega_{s}}e_{qs}^{\prime }-\frac{1}{{T_{r}\omega }_{s}}e_{ds}^{\prime }-\upsilon_{qs}+\frac{L_{m}}{L_{rr}}\upsilon_{qr})\\ \frac{di_{ds}}{dt}=\frac{\omega_{b}}{L_{s}^{\prime }}(-\omega_{s}L_{s}^{\prime }i_{qs}-R_{1}i_{qs}+\frac{\omega_{r}}{\omega_{s}}e_{ds}^{\prime }+\frac{1}{{T_{r}\omega }_{s}}e_{qs}^{\prime }-\upsilon_{ds}+\frac{L_{m}}{L_{rr}}\upsilon_{qr})\\ \frac{de_{qs}^{\prime }}{dt}=\omega_{b}\omega_{s}[R_{2}i_{ds}-\frac{1}{{T_{r}\omega }_{s}}e_{qs}^{\prime }+(1-\frac{\omega_{r}}{\omega_{s}})e_{ds}^{\prime }-\frac{L_{m}}{L_{rr}}\upsilon_{dr}]\\ \frac{de_{ds}^{\prime }}{dt}=\omega_{b}\omega_{s}[-R_{2}i_{qs}-(1-\frac{\omega_{r}}{\omega_{s}})e_{qs}^{\prime }-\frac{1}{{T_{r}\omega }_{s}}e_{ds}^{\prime }+\frac{L_{m}}{L_{rr}}\upsilon_{qr}] \end{array}\right.$$ (7)

where *T*_r_ represents the time constant of rotor; *R*_1_ and *R*_2_ mean the equivalent resistance on the stator side and rotor side respectively; $L_{s}^{\prime },$ *L*_ss_, *L*_rr_ and *L*_m_ denote the equivalent inductance on the stator side, stator inductance, rotor inductance and mutual inductance respectively. Other parameters can be referred to literature [32].

The electromagnetic torque *T*_e_ generated by the DFIG is described as

$$T_{e}=(e_{qs}^{\prime }/\omega_{s})i_{qs}+(e_{ds}^{\prime }/\omega_{s})i_{ds}$$ (8)

The shaft system can be simply described as a single lumped-mass system with a lumped inertia constant *H*_m_, as follows

$$H_{m}=H_{t}+H_{g}$$ (9)

where *H*_t_ and *H*_g_ denote two inertia constants of wind turbine and DFIG, respectively.

The electromechanical dynamics is then computed by

$$\frac{d\omega_{m}}{dt}=\frac{1}{2H_{m}}(T_{m}-T_{e}-D\omega_{m})$$ (10)

where *ω*_m_ denotes lumped-mass system’s rotational speed, which meets *ω*_m_ = *ω*_r_; *D* = 0.05 p.u. means lumped system’s damping; and *T*_m_ stands for the mechanical torque, i.e., *T*_m_ = *P*_m_/*ω*_m_.

### 2.3 VSC-HVDC system model

As demonstrated in Fig 1, VSC-HVDC system model can be described as follows [33,34]:

$$\left\{ \begin{array}{l} L_{dc}\frac{di_{s1}}{dt}=-R_{dc}i_{s1}+(U_{d1}-U_{d2})\\ i_{s1}=-i_{s2}\\ C_{d1}\frac{dU_{d1}}{dt}={i_{d1}-i}_{s1}\\ C_{d2}\frac{dU_{d2}}{dt}={i_{d2}-i}_{s2} \end{array}\right.$$ (11)

where *i*_d1_ and *i*_d2_ represent DC currents of VSC on both sides; *U*_d1_ and *U*_d2_ denote DC voltages of VSC on both sides; *C*_d1_ and *C*_d2_ mean DC capacitances of VSC on both sides.

## 3. CBAS algorithm

### 3.1 BAS algorithm

BAS algorithm is a novel biology-based meta-heuristic algorithm, which is mainly based on special food detecting and searching behaviour of long-horn beetles characterized by extremely long antennae in nature [29]. Such long antennae are a very common symbol in most beetle species, and it is composed of various types of olfactory receptor cells. The main function of large antennae is to expand detection range, within this range, beetles can better capture the odour of prey and detect sex pheromones that may be suitable for mating [29]. Basically, beetle uses two antennae to randomly detect nearby areas, and the detection direction depends on which side has a higher odour.

In BAS algorithm, at the *k*th time, the location of each beetle is considered as a vector *x*^*k*^ (*k* = 1,2,…). Meanwhile, the fitness function is represented by *f*(*x*), which means odour concentration locates at *x*, while its maximum value directly relies on where odour begins to diffuse, called source point. Inspired by stochastic searching mechanism of beetles, two stages are mainly contained, namely, searching and detecting.

(a) *Searching*: Stochastic searching direction of beetles is defined by

$$\vec{b}=\frac{rnd(D_{dim},1)}{\left||rnd(D_{dim},1)|\right|}$$ (12)

where rnd(.) means a stochastic function and *D*_dim_ stands for location dimensions, respectively.

Besides, for more accurately replicating actual searching behaviour of beetle’s antennae, right-hand and left-hand searching behaviours are adopted, as follows:

$$x_{r}=x^{k}+d^{k}\vec{b}$$ (13)

$$x_{l}=x^{k}-d^{k}\vec{b}$$ (14)

where *x*_r_ and *x*_l_ denote location in the right-hand and left-hand searching area, respectively; and *d* is sensing length of antennae, which initial value should be large enough to avoid premature convergence at the initial phase, and decreases over time.

(b) *Detecting*: An iterative model is presented which takes both odour detection and searching behaviour into consideration, as follows:

$$x^{k}=x^{k-1}+\delta^{k}\vec{b}sign(f(x_{r})-f(x_{l}))$$ (15)

where *δ* denotes step size that indicates convergence rate, while initialization of *δ* and searching area should be equal; and sign(.) means sign function, respectively.

Particularly, the updating rule of parameters which directly influences searching behaviour, e.g., antennae length *d* and step size *δ*, can be expressed as follows:

$$d^{k}=0.95d^{k-1}+0.01$$ (16)

$$\delta^{k}=0.95\delta^{k-1}$$ (17)

### 3.2 CBAS algorithm

#### 3.2.1 Cooperative group

**Fig 2:**
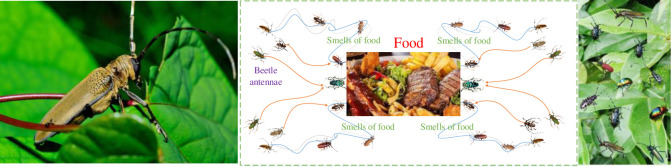
Optimization principle of CBAS algorithm.

BAS algorithm only adopts a single beetle to seek a potentially better solution, which is easy to fall into local optimums. In order to overcome such drawbacks, CBAS algorithm employs a cooperative group with multiple beetles to find potential better solutions, as demonstrated in Fig 2. Hence, CBAS algorithm not only contains a detecting stage (i.e., global search) like BAS algorithm, but also a local searching behavior to approximate the current best solution, which can be described by

$$x_{i}^{k}=x_{i}^{k-1}+{\omega_{1}^{k}∙\delta }^{k}∙\vec{b}sign(f(x_{ir})-f(x_{il}))+\omega_{2}^{k}∙C∙r_{1}(x_{best}^{k-1}-x_{i}^{k-1})$$ (18)

where subscript *i* means the *i*th beetle; $\omega_{1}^{k}$ and $\omega_{2}^{k}$ represent dynamic weights of global exploration and local exploitation, respectively; *C* stands for a constant coefficient; *r*_1_ is a stochastic value from [0, 1]; and $x_{best}^{k-1}$ denotes current best solution until the (*k*-1)th iteration.

#### 3.2.2 Dynamic balance between local exploitation and global exploration

Like other meta-heuristic algorithms, it is significant to achieve a stable and desirable optimization of a dynamic balance between local exploitation and global exploration. For example, if CBAS algorithm attaches more attention to local exploitation, it will easily result in a low-quality local optimum; otherwise, it will result in a low optimization efficiency to seek a better solution. In order to realize a dynamic balance between local exploitation and global exploration, weights in Eq (18) are designed to be time-varying as iteration increases, yields

$$\omega_{1}^{k}=\omega_{min}+\left(1-\frac{k}{k_{max}}\right)∙(\omega_{max}-\omega_{min})$$ (19)

$$\omega_{2}^{k}=1-\omega_{1}^{k}$$ (20)

where *k*_max_ means maximum iteration number; *ω*_max_ and *ω*_min_ denote the maximum and minimum weights, respectively.

Note that global exploration weight $\omega_{1}^{k}$ will gradually decrease as iteration number grows based on Eq (19), while local exploitation weight $\omega_{2}^{k}$ will gradually increase since their sum is equal to be 1 in Eq (20). According to such improvement, global exploration ability of CBAS algorithm can be significantly improved in initial optimization stage, which can effectively boost searching efficiency and probability of high-quality solutions. As iteration number increases, CBAS algorithm tends to concentrate on local exploitation, which can further improve solution quality.

Furthermore, parameters of BAS algorithm, including antennae length *d* and step size *δ*, are prone to considerably decrease with an exponential type in Eqs (16) and (17), upon which a broad global exploration cannot be achieved smoothly. To remedy such problem, an exponential reduction is displaced by a linear reduction in CBAS algorithm, as follows:

$$d^{k}=\left(1-\frac{k}{k_{max}}\right)∙d_{max}$$ (21)

$$\delta^{k}=\left(1-\frac{k}{k_{max}}\right)∙\delta_{max}$$ (22)

where *d*_max_ and *δ*_max_ denote the maximum antennae length and maximum step size, respectively.

### 3.3 Optimization process

**Fig 3:**
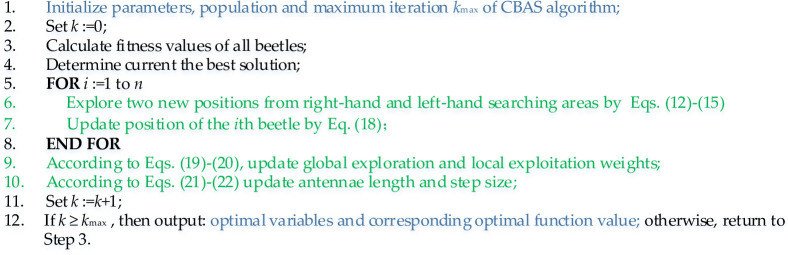
Optimization process of CBAS algorithm.

In general, the optimization process of CBAS algorithm is given in Fig 3.

## 4. CBAS algorithm design for hybrid AC/VSC-HVDC system integrated with DFIG

### 4.1 Control design of synchronous generator

**Fig 4:**
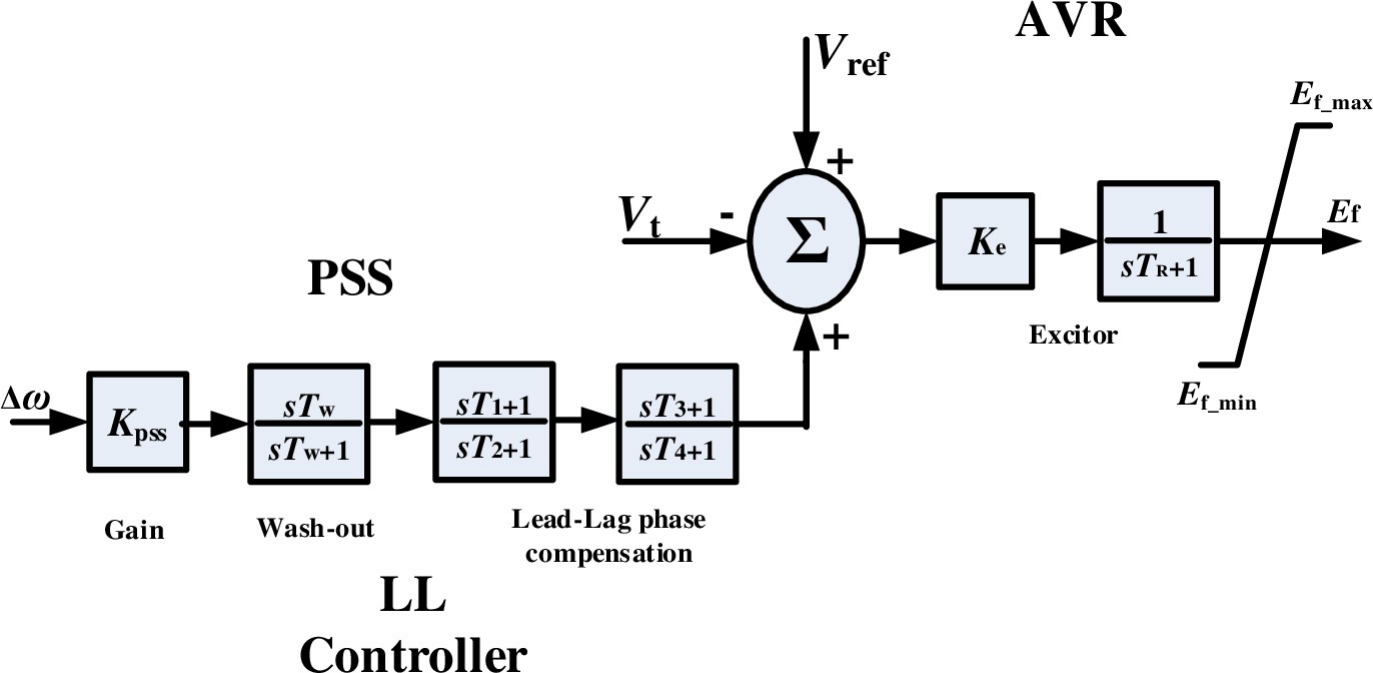
The conventional LL+AVR controller of synchronous generator structure.

Fig 4 shows the conventional lead-lag (LL)+ automatic voltage regulator (AVR) controller of synchronous generator structure, the dynamic model of AVR and excitor can be expressed as follows:

$${\dot{E}}_{f}=\frac{K_{e}(V_{ref}-V_{t}+U_{pss})}{T_{R}}-E_{f}/T_{R}$$ (23)

where *E*_f_ denotes excitor voltage; *V*_ref_ represents voltage reference; *V*_*t*_ means synchronous generator terminal voltage; *U*_pss_ represents the voltage of PSS; *K*_e_ = 200 denotes excitor gain; *T*_R_ = 0.01 stands for the time constant of excitor.

The transfer function of PSS can be described as follows [34]:

$$U_{PSS}(s)=K_{PSS}\left[\frac{T_{w}s}{1+T_{w}s}\right]\left[\frac{1+T_{1}s}{1+T_{2}s}\right]\left[\frac{1+T_{3}s}{1+T_{4}s}\right]\Delta \omega (s)$$ (24)

where *K*_PSS_ denotes PSS gains; *T*_w_ = 10 represents the time constant of wash-out process; *T*_1_ and *T*_2_ denote two first-order time constants of lead-lag phase; *T*_3_ and *T*_4_ denote two second-order time constants of lead-lag phase; Δ*ω* stands for rotor speed difference.

### 4.2 Control design of DFIG

The power of DFIG can be described as follows

$$\left\{ \begin{array}{c} P_{s}=V_{ds}I_{ds}+V_{qs}I_{qs}=V_{ds}I_{ds}\\ Q_{s}=V_{qs}I_{ds}-V_{ds}I_{qs}=-V_{ds}I_{qs} \end{array}\right.$$ (25)

**Fig 5:**
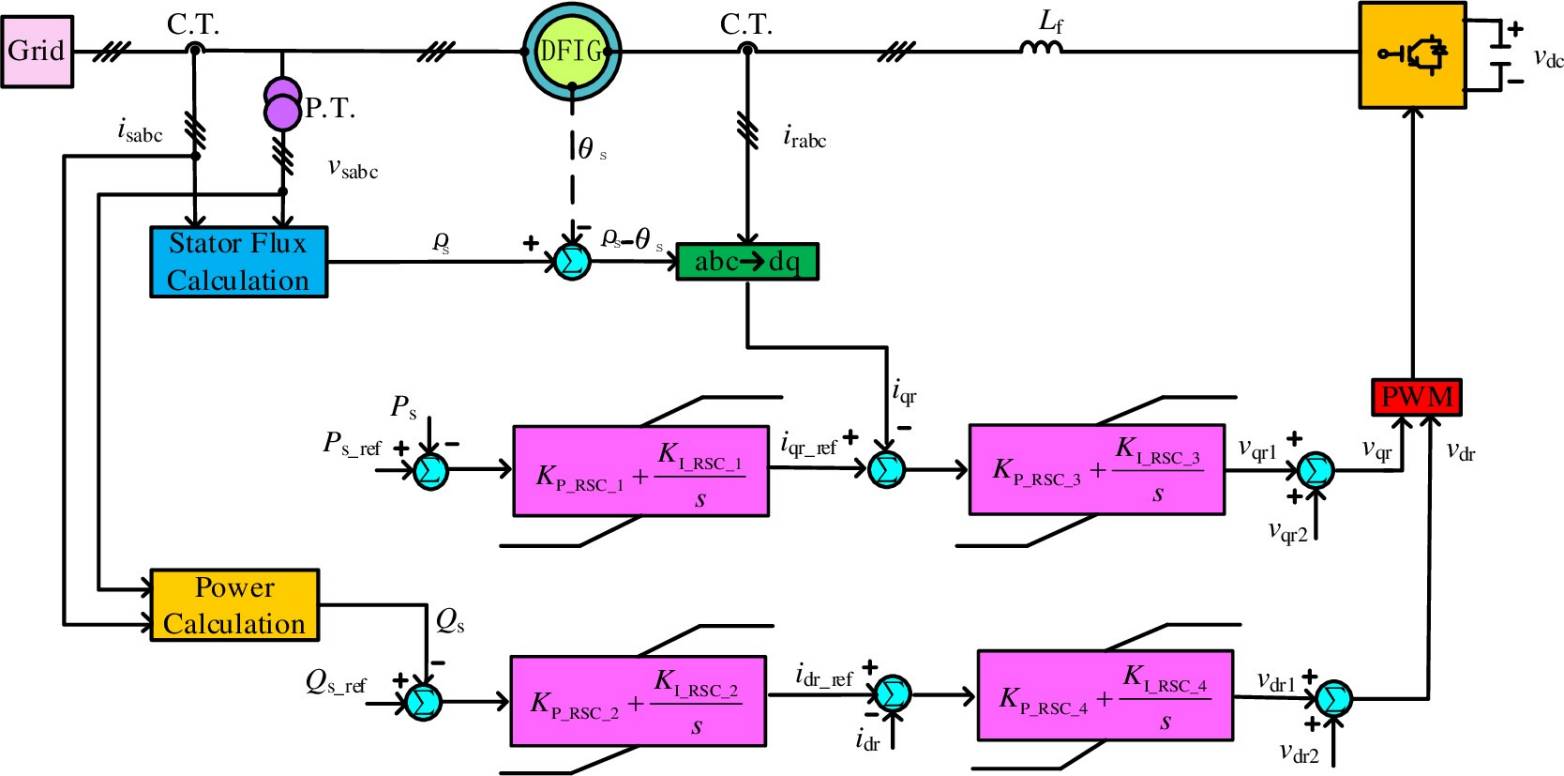
The overall control structure of DFIG.

Control design of rotor side converter (RSC) of DFIG is the major task, in which outer control loops are utilized for regulation of DFIG active and reactive power independently. In particular, two currents related to the compensation terms *v*_qr2_ and *v*_dr2_ are regulated to acquire the final controller outputs *v*_qr_ and *v*_dr_ in inner control loops. Based on this operation framework, four interactive PI loops are used to obtain the optimal control performance, as shown in Fig 5, which corresponding symbols can be expressed as [32]

$$\left\{ \begin{array}{c} s=\frac{\omega_{s}-\omega_{r}}{\omega_{s}}\\ \sigma =1-\frac{L_{m}^{2}}{L_{s}L_{r}}\\ i_{ms}=\frac{v_{qs}-R_{s}i_{qs}}{\omega_{s}L_{m}}\\ v_{qr2}=s\omega_{s}(\sigma L_{r}i_{dr}+\frac{L_{m}^{2}i_{ms}}{L_{s}})\\ v_{dr2}=-s\omega_{s}\sigma L_{r}i_{qr} \end{array}\right.$$ (26)

where *s* denotes the DFIG slip and *σ* represents the leakage coefficient.

### 4.3 Control design of VSC-HVDC system

**Fig 6:**
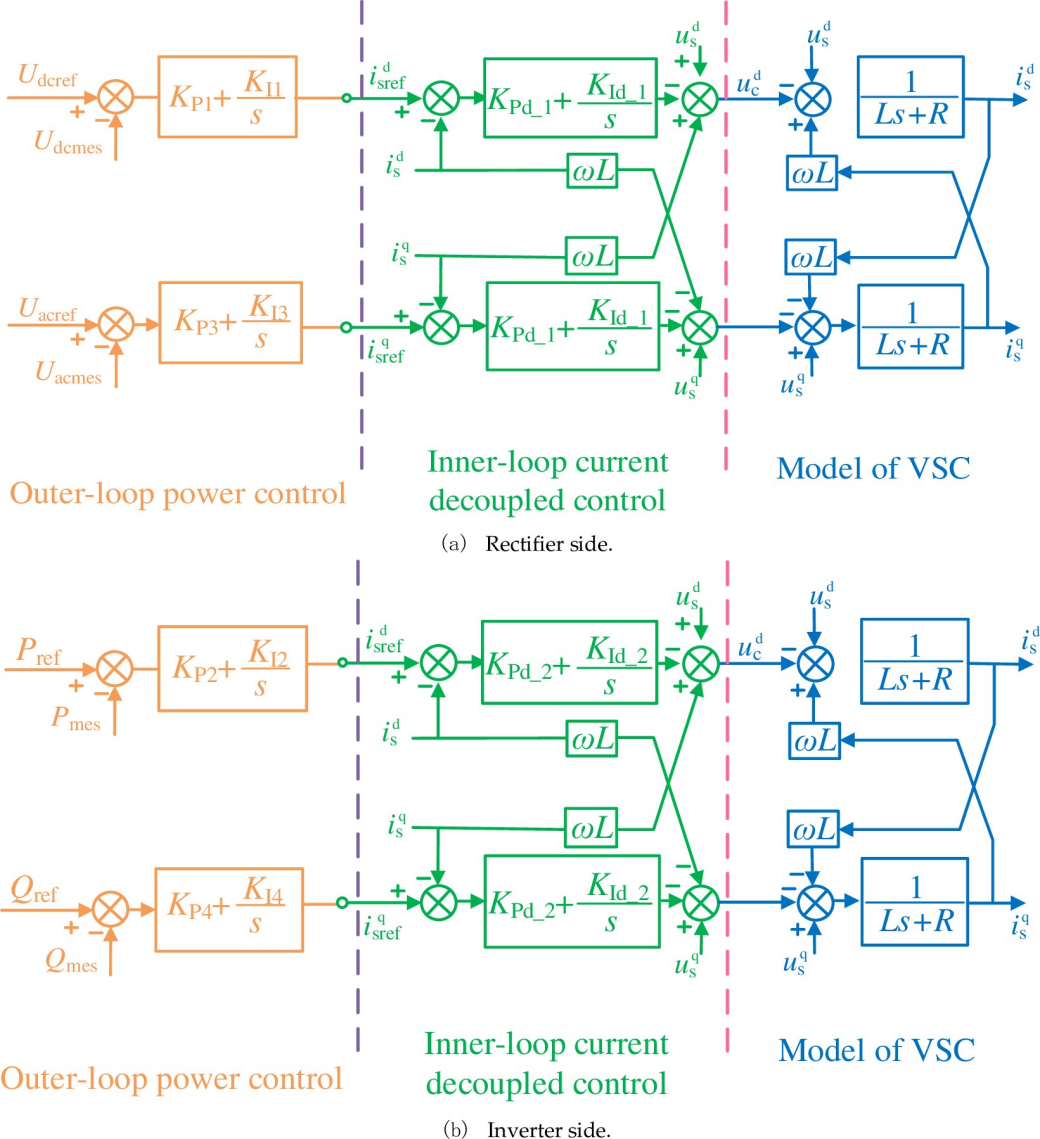
Control structure of VSC-HVDC system.

Here, rectifier side adopts outer-loop control of constant AC voltage and constant DC voltage regulation, while inverter side adopts outer-loop control of constant active power and constant reactive power regulation [35], which is illustrated in Fig 6.

### 4.4 Optimal coordinated controller gains optimization

Here, CBAS algorithm is utilized to optimize PSS gains of synchronous generator *K*_PSS_, *T*_1_, *T*_2_, *T*_3_ and *T*_4_, as well as PI controller gains of VSC-HVDC system and RSC of DFIG, namely, *K*_P1_, *K*_I1_, *K*_P2_, *K*_I2_, *K*_P3_, *K*_I3_, *K*_P4_, *K*_I4_, *K*_Pd_1_, *K*_Id_1_, *K*_Pd_2_, *K*_Id_2_, *K*_P_RSC_1_, *K*_I_RSC_1_, *K*_P_RSC_2_, *K*_I_RSC_2_, *K*_P_RSC_3_, *K*_I_RSC_3_, *K*_P_RSC_4_ and *K*_I_RSC_4_. In order to realize an optimal control performance, the above gains are adjusted under the following three typical operating conditions, e.g., (a) three-phase short-circuit fault, (b) load disconnection, and (c) DFIG loss. Moreover, the objective function of the designed system is expressed as

$$Minimizef(x)=\sum_{Three\;cases}W_{k}\int_{0}^{T}\left(|\delta_{ij}-\delta_{ij}^{*}|+|\omega_{ij}-\omega_{ij}^{*}|\right)dt$$

$$subject\;to\;\left\{ \begin{array}{c} {0\leq K}_{P1}\leq 400\\ {0\leq K}_{I1}\leq 10\\ {0\leq K}_{P2}\leq 5\\ {0\leq K}_{I2}\leq 150\\ {-400\leq K}_{P3}\leq 0\\ {-10\leq K}_{I3}\leq 0\\ {0\leq K}_{P4}\leq 400\\ {0\leq K}_{I4}\leq 5\\ 0{\leq K}_{Pd\_1}\leq 5\\ 0{\leq K}_{Id\_1}\leq 5\\ 0{\leq K}_{Pd\_2}\leq 0.1\\ 0\leq K_{Id\_2}\leq 0.5\\ {0\leq K}_{PSS}\leq 20\\ 0\leq T_{1}\leq 2\\ {0\leq T}_{2}\leq 1\\ {0\leq T}_{3}\leq 5\\ 0\leq T_{4}\leq 5\\ 0\leq K_{P\_RSC\_i}\leq 1\\ 0\leq K_{I\_RSC\_i}\leq 10 \end{array}\right.\;k=1,2,3;i,j=1,2,3,4\;and\;i\neq j$$ (27)

where *W*_*k*_ represents corresponding weight coefficient under each operation condition, which are set as 0.3, 0.5 and 0.2, respectively; *δ*_*ij*_ and $\delta_{ij}^{*}$ denote rotor angle difference and its reference of generator #*i* and #*j*; *ω*_*ij*_ and $\omega_{ij}^{*}$ represent rotor speed difference and its reference of generator #*i* and #*j*; Simulation time *T* = 120 s.

### 4.5 Overall control flow for hybrid AC/VSC-HVDC system integrated with DFIG

**Fig 7:**
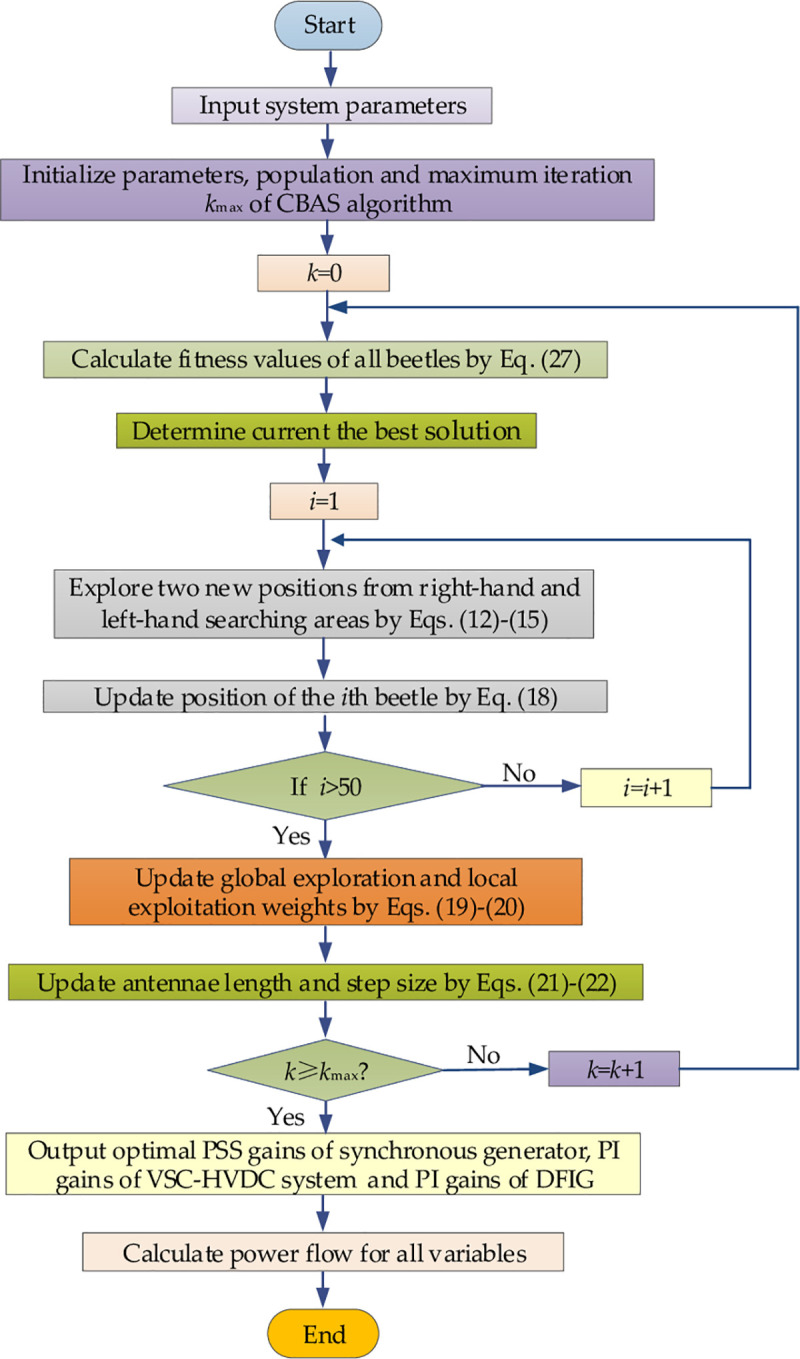
Overall control flow for hybrid AC/VSC-HVDC system integrated with DFIG.

To this end, overall control flow of CBAS algorithm for hybrid AC/VSC-HVDC system integrated with DFIG is shown in Fig 7.

## 5. Case studies

In this section, control performance of CBAS algorithm in hybrid AC/VSC-HVDC system integrated with DFIG is compared to that of manual tuning, particle swarm optimization (PSO) algorithm [36], GA [37], and BAS algorithm [29] under the above cases. Note that all approaches are executed in 10 independent runs to acquire statistical results and convergence graphs [38,39], while the best solutions are used as the optimal gains. In addition, AC system frequency is set as 50 Hz and parameters of hybrid AC/VSC-HVDC system integrated with DFIG are tabulated in Table 1 while algorithm parameters are given in Table 2. Besides, ode23 was selected as the solver, and the sampling rate was set to 0.001 s.

**Table 1:** System parameters.

**Synchronous generator No. (in p.u.)**	*x*_d*i*_	$x_{di}^{\prime }$	$T_{d0i}^{\prime }$	*H*_*i*_	*D*_*i*_
**1,2,4**	1.8	0.3	8.0	13	0
**Wind turbine**	*ρ*	*R*	*v*_wind_	*λ*_opt_	*H*_m_
	1.225kg/m^3^	58.59m^2^	12m/s	6.325	4.4s
**DFIG (in p.u.)**	*R*_s_	*L*_m_	*R*_r_	*L*_ss_	*L*_*rr*_
**3**	0.005	4.0	1.1*R*_s_	1.01*L*_m_	1.005*L*_ss_
	$L_{s}^{\prime }$	*T*_r_	*R*_1_	*R*_2_	
	$L_{ss}-L_{m}^{2}/L_{rr}$	*L*_*rr*_/*R*_r_	*R*_s_+*R*_2_	(*L*_m_/*L*_*rr*_)^2^*R*_r_	
**Transformer (in p.u.)**	Resistance			Impedance	
	0.0025			0.0167	
**Line No. (in p.u.)**	Resistance			Impedance	
**1, 2**	0.0025			0.025	
**3, 4**	0.001			0.01	
**5–10**	0.005			0.2245	
**Load No. (in p.u.)**	Active power			Reactive power	
**1**	9.67			-1	
**2**	18.67			-2.5	
**VSC (in p.u.)**	Resistance			Impedance	
	0.005			0.2245	
**AC system base voltage**	*u*_ACbase_			132kV	
**DC cable base voltage**	*u*_DCbase_			150kV	
**System base power**	*S*_base_			100MVA	

**Table 2:** Parameters of four algorithms.

Algorithm	Parameters			
**PSO**	Accelerated constant	Speed range	Population	*k*_max_
	1.49445	[-0.5, 0.5]	50	20
**GA**	Crossover rate	Mutation rate	Population	*k*_max_
	0.8	0.1	50	20
**BAS**	Step size	Sensing diameter	Population	*k*_max_
	0.9	0.9	50	20
**CBAS**	Step size	Sensing diameter	Population	*k*_max_
	0.9	0.9	50	20

**Fig 8:**
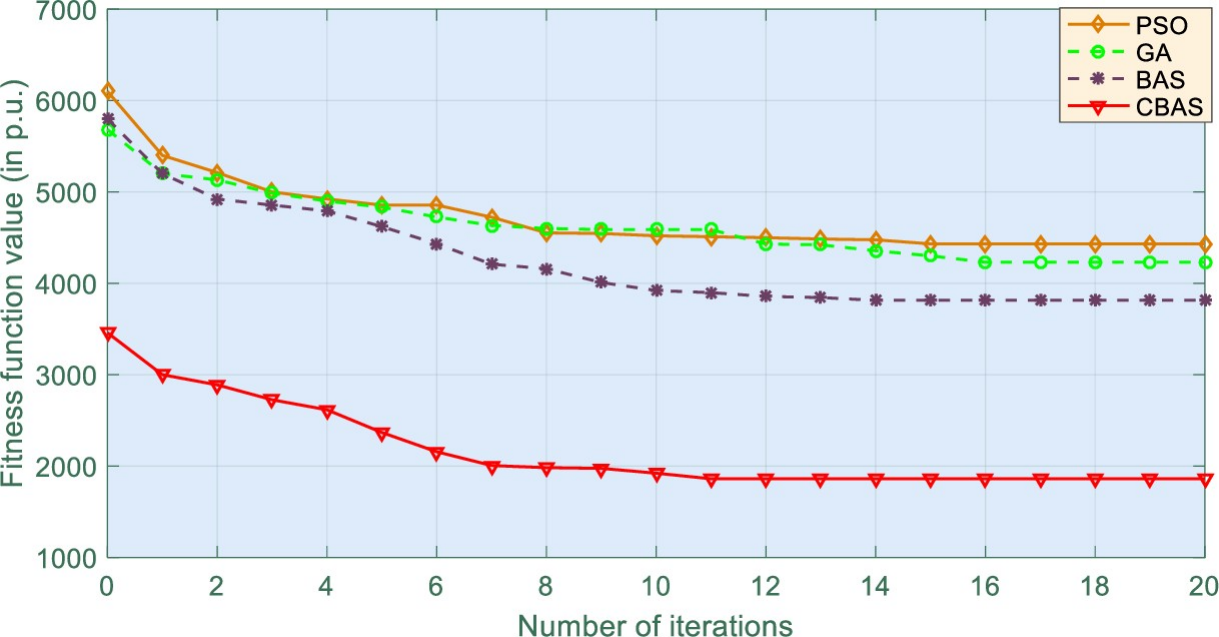
Comparison of the best convergence performance of four algorithms obtained in 10 runs.

**Fig 9:**
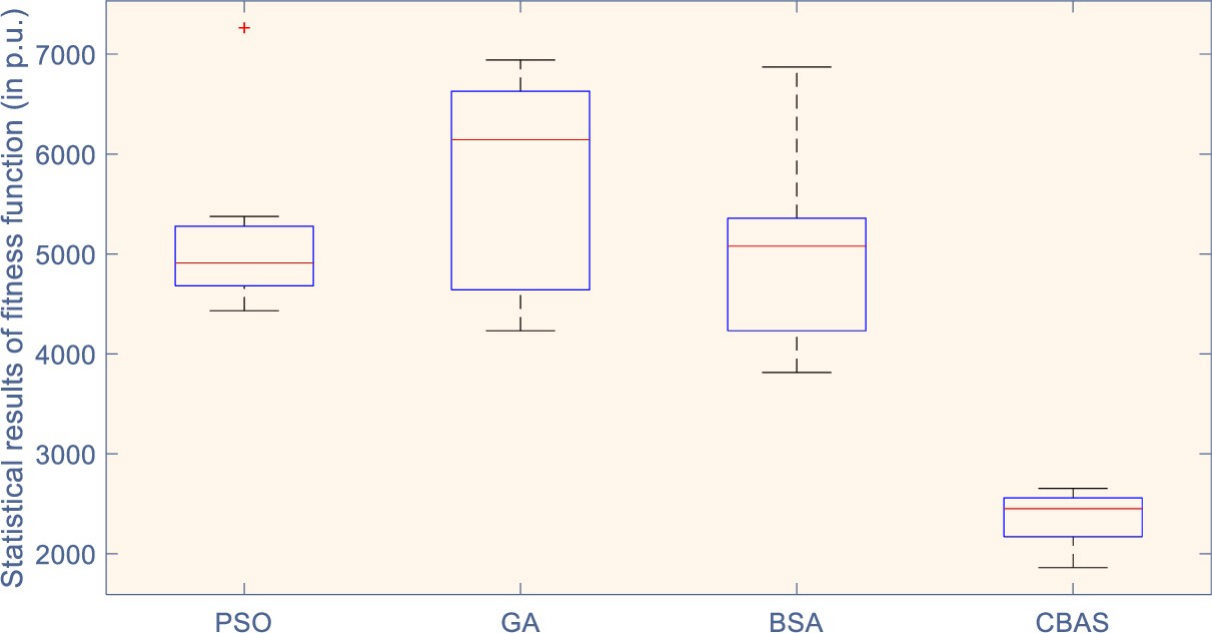
Statistical results of fitness function acquired in 10 runs (in p.u.).

Moreover, convergence of four algorithms is shown in Fig 8, which indicates that CBAS algorithm owns the fastest convergence under all three evaluation indices. Fig 9 illustrates boxplot of different methods, i.e., distribution of simulation results, which shows that CBAS algorithm can distribute within the smallest range with minimal lower and upper bounds among all algorithms. It verifies that CBAS algorithm owns the highest convergence stability and searching ability. As a result, CBAS algorithm can effectively avoid local optimum trapping. Furthermore, convergence rate of CBAS algorithm can be considerably improved by its multiple beetles based cooperative searching mechanism. At last, the optimized control gains are tabulated in Table 3.

**Table 3:** Optimized controller gains.

Algorithm Controller		Manual tuning	PSO	GA	BAS	CBAS
**PSS of synchronous generator**	*K*_PSS_	8.969	10.157	5.621	5.954	8.672
	*T*_1_	0.01	0.216	1.755	1.491	0.226
	*T*_2_	0.237	0.126	0.449	0.302	0.148
	*T*_3_	1.568	2.162	1.265	2.556	0.234
	*T*_4_	0.704	0.157	2.238	0.759	0.114
RSC of DFIG	*K*_P_RSC_1_	0.234	0.510	0.866	0.643	0.422
	*K*_I_RSC_1_	8.996	6.254	3.987	5.226	4.598
	*K*_P_RSC_2_	0.436	0.911	0.538	0.294	0.198
	*K*_I_RSC_2_	7.357	5.694	8.327	5.102	3.269
	*K*_P_RSC_3_	0.369	0.128	0.567	0.912	0.248
	*K*_I_RSC_3_	7.356	6.982	4.328	2.397	5.264
	*K*_P_RSC_4_	0.346	0.289	0.861	0.674	0.423
	*K*_I_RSC_4_	3.259	8.256	4.336	6.196	4.233
**Rectifier of VSC-HVDC system**	*K*_I1_	7.355	5.068	9.561	8.636	9.048
	*K*_P1_	325.153	382.077	306.369	379.032	361.342
	*K*_I3_	-6.255	-5.066	-7.121	-7.484	-7.221
	*K*_P3_	-300.54	-382.076	-390.453	-357.35	-317.498
	*K*_Pd_1_	2.548	1.262	2.648	1.949	1.828
	*K*_Id_1_	2.687	1.841	1.194	4.264	3.383
**Inverter of VSC-HVDC system**	*K*_I2_	57.156	100.154	140.278	85.161	88.636
	*K*_P2_	3.169	1.165	3.437	4.392	2.041
	*K*_I4_	2.144	1.681	4.851	0.513	0.873
	*K*_P4_	300.195	230.263	250.215	180.248	150.441
	*K*_Pd_2_	0.087	0.047	0.095	0.054	0.093
	*K*_Id_2_	0.371	0.285	0.414	0.295	0.474

### 5.1 Three-phase short-circuit fault

**Fig 10:**
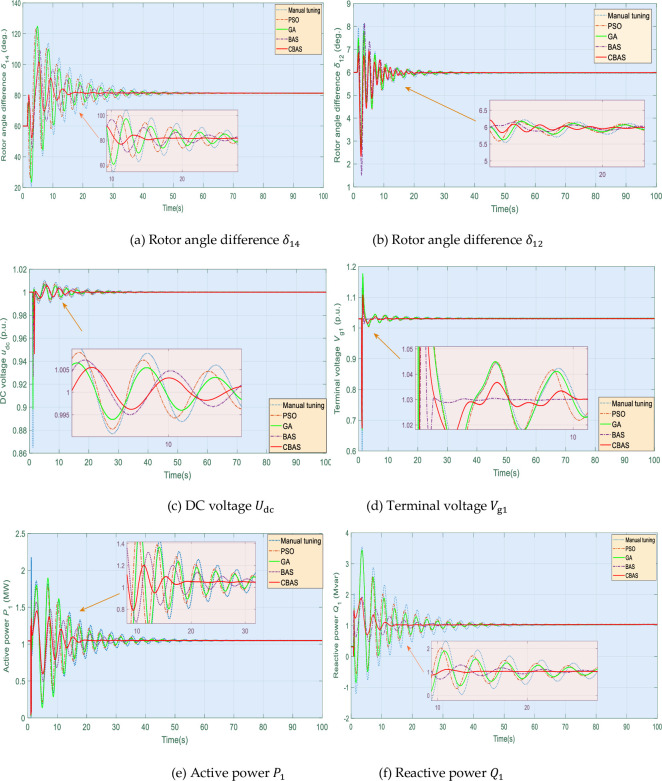
System responses acquired under the three-phase short-circuit fault.

To validate control performance of CBAS algorithm under varying operation conditions, a three-phase short-circuit fault occurs on the middle of transmission line 7 when *t* = 1s, and removed at 1.1s, as illustrated in Fig 1. Besides, as shown in Fig 10, simulation results of corresponding system responses can explicitly validate that CBAS algorithm can suppress the power oscillation most effectively and efficiently. In contrast, manual tuning reveals the largest overshoot of active power and the slowest convergence rate compared to that of other algorithms.

### 5.2 Load disconnection

**Fig 11:**
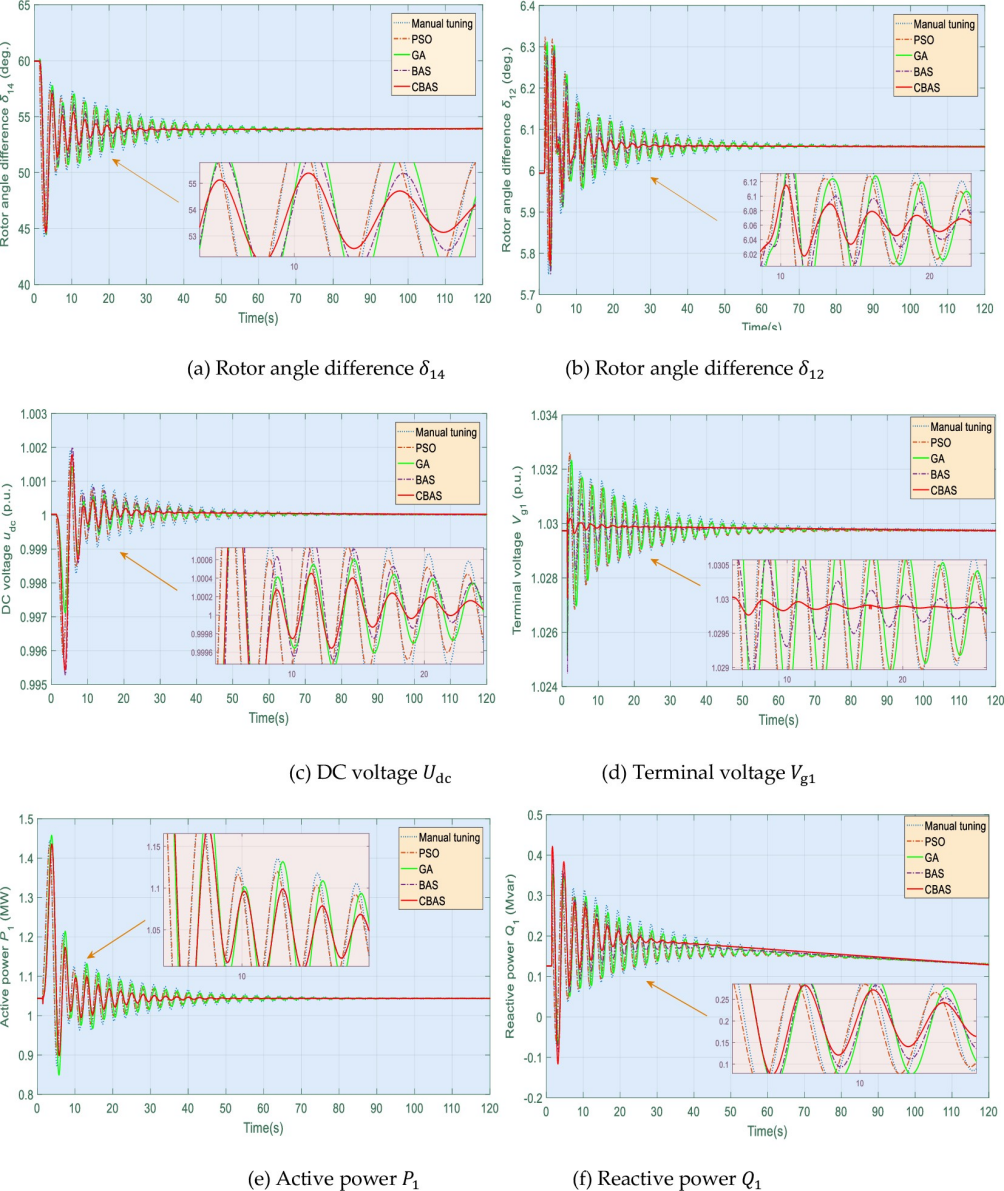
System responses obtained under load disconnection.

This test aims to investigate the effectiveness and reliability of various controllers under load disconnection. Hence, load 1 is disconnected when *t* = 1s (highlighted in Fig 1), while Fig 11 depicts corresponding system responses. Here, BAS algorithm can hardly maintain an effective control performance because single beetle searching strategy easily falls into a local optimum, along with slow convergence speed. In contrast, CBAS algorithm can compensate active/reactive power imbalance with the highest tracking speed and the lowest tracking error compared to that of other algorithms. Besides, tracking results of rotor angle difference *δ* also demonstrate that CBAS algorithm can stably and rapidly restore the disturbed system compared against other approaches based on its cooperative searching mechanism to maintain proper balance between local exploitation and global exploration.

### 5.3 DFIG loss

**Fig 12:**
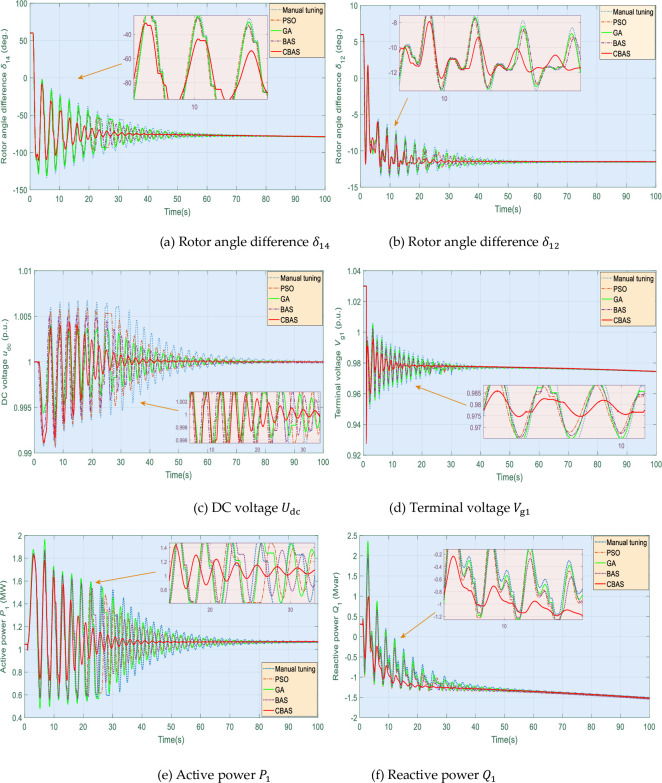
System responses obtained under DFIG loss.

In this case, severe power oscillation is caused by DFIG loss when *t* = 1s, which is usually caused by the internal failure of the generator (such as damage to the mechanical parts of the generator leading to the start of the protection device), while corresponding system responses are presented in Fig 12, which illustrates that all algorithms are subjected to such active power oscillations. However, CBAS algorithm can effectively suppress such malignant oscillations as it can adjust rotor angle difference with the slightest overshoot and highest convergence speed.

### 5.4 Comparative analysis

Table 4 reveals that CBAS algorithm owns the fastest convergence rate while Table 5 shows that CBAS algorithm owns the minimum fitness function in 10 runs. Lastly, the integral of absolute error (IAE) [40–42] of each algorithm in three scenarios are given by Table 6, in which ${IAE}_{x}=\int_{0}^{T}\left|x-x^{*}\right|dt$ and x* denotes the reference of variable *x*, respectively [43,44]. In particular, IAE_*δ*_ of CBAS algorithm is merely 32.28%, 55.41%, 48.81%, and 56.94% of that of manual tuning [45–47], PSO, GA, and BAS algorithm, respectively (bold colour indicates the best results in Tables 4–6).

**Table 4:** Statistical results of convergence performance acquired by four algorithms in 10 runs.

Algorithm	Execution time (hours)			Convergence time (hours)			Iteration number		
	Max.	Min.	Mean	Max.	Min.	Mean	Max.	Min.	Mean
**PSO**	3.541	1.165	2.549	3.186	0.873	2.109	18	15	16.547
**GA**	3.236	1.512	2.274	2.075	1.209	1.835	17	16	16.135
**BAS**	3.164	1.015	2.141	2.848	0.711	1.625	18	14	15.183
**CBAS**	**1.154**	**0.714**	**0.894**	**0.808**	**0.393**	**0.556**	**14**	**11**	**12.436**

**Table 5:** Statistical results of fitness function acquired by four algorithms in 10 runs (in p.u.).

Algorithm	Maximum	Minimum	Mean
**PSO**	7263.154	4432.132	5133.164
**GA**	6941.324	4231.512	5741.236
**BAS**	6871.642	3814.264	4943.345
**CBAS**	**2654.365**	**1861.165**	**2364.146**

**Table 6:** IAE indices of five algorithms acquired in three cases (in p.u.).

Cases	IAE indices	Manual tuning	PSO	GA	BAS	CBAS
**Three-phase short-circuit fault**	IAE_*δ*_	1.27	0.74	0.84	0.72	**0.41**
**Load disconnection**	IAE_*δ*_	4.54×10^−2^	2.84×10^−2^	2.62×10^−2^	2.14×10^−2^	**5.82×10**^**−4**^
**DFIG loss**	IAE_*δ*_	3.28	1.49	1.54	1.35	**0.97**

## 6. Conclusions

This paper designs a novel CBAS algorithm for an optimal coordinated control of Hybrid AC/VSC-HVDC system integrated with DFIG, which owns the following three contributions/novelties:

1. Compared to original BAS algorithm, CBAS algorithm can remarkably improve optimization efficiency via a cooperative group of multiple beetles instead of a single beetle. Besides, it can also acquire a high-quality optimum through a dynamic and proper balance between local exploitation and global exploration; In addition, the convergence time of CBAS algorithm is only 55.27% of BAS algorithm;
2. CBAS algorithm is utilized to optimally tune the interacted PSS gains of synchronous generator, PI gains of VSC-HVDC system, and PI gains of DFIG, such that the overall power oscillations can be minimized under various operation conditions;
3. Case studies validate that CBAS algorithm can achieve active power demand variation tracking with the highest tracking speed and lowest tracking error. Moreover, it can also effectively and efficiently restore the disturbed system. Statistical results of fitness function further verify that CBAS algorithm can find the best quality optimum with the highest convergence stability and reliability compared to that of manual tuning and other meta-heuristic algorithms. Particularly, the average results of fitness function acquired by CBAS algorithm is merely 46.05%, 41.18%, and 47.82% of that of PSO, GA, and BAS algorithm, respectively.

Besides, three future studies are given as follows:

1. Hardware experiment should be undertaken to validate the feasibility;
2. Advanced methods are encouraged to be constructed to overcome system uncertainties;
3. More load models, e.g., electric vehicle load need to be considered.

## Abbreviations

**Table:** Nomenclature.

*δ*_*i*_	generator rotor angle	*x*_r_, *x*_l_	location in right-hand and left-hand searching area
*ω*_*i*_	generator rotor speed	$\omega_{1}^{k}$, $\omega_{2}^{k}$	dynamic weights of global exploration and local exploitation
*R*_1_	equivalent resistance of coupling transformer	$x_{best}^{k-1}$	current best solution until the (*k*-1)th iteration
*R*_2_	equivalent resistance of phase reactor	*d*_max_, *δ*_max_	maximum antennae length and maximum step size
*R*_dc_, *L*_dc_	resistance and inductance of DC line	*W*_*i*_	weight coefficient under a certain operation condition
*I*_d*i*_, *I*_q*i*_	*d*-axis and *q*-axis generator current	*δ*_*ij*_	rotor angle difference of generator *i* and *j*
*i*_s*i*_	current flowing from AC grid side to VSC	$\delta_{ij}^{*}$	reference value of *δ*_*ij*_
*Y*_*ij*_	equivalent admittance between the *i*th and *j*th nodes	*k*_max_	maximum iteration number
*G*_*ii*_	equivalent self-conductance of the *i*th machine	***Abbreviations***	
*E*_f_	excitor voltage	DFIG	doubly-fed induction generator
*U*_pss_	voltage of PSS	PID	proportional-integral-differential
*K*_e_	excitor gains	CBAS	cooperative beetle antenna search
*T*_R_	time constant of excitor	ITLO	interactive teaching-learning optimizer
*T*_w_	time constant of wash-out	PSS	power system stabilizer
*i*_d1_, *i*_d2_	DC currents	LL	lead-lag
*C*_d1_, *C*_d2_	DC capacitances	AVR	automatic voltage regulator
