# Correction: Optimal coordinated control of hybrid AC/VSC-HVDC system integrated with DFIG via cooperative beetle antennae search algorithm

**DOI:** 10.1371/journal.pone.0244757

**Published:** 2020-12-23

**Authors:** The PLOS ONE staff

The following information is missing from the Funding statement: This study was supported by the National Key R&D Program of China, 2016YFB0900600 [J.Hao] and the Technology Projects of State Grid Corporation of China, 52094017000W [J.Huang]. The publisher apologizes for the error.
